# The First Step of Biodegradation of 7-Hydroxycoumarin in *Pseudomonas mandelii* 7HK4 Depends on an Alcohol Dehydrogenase-Type Enzyme

**DOI:** 10.3390/ijms22041552

**Published:** 2021-02-04

**Authors:** Arūnas Krikštaponis, Gintaras Urbelis, Rolandas Meškys

**Affiliations:** 1 Department of Molecular Microbiology and Biotechnology, Institute of Biochemistry, Life Sciences Center, Vilnius University, Sauletekio al. 7, LT-10257 Vilnius, Lithuania; rolandas.meskys@bchi.vu.lt; 2 Department of Organic Chemistry, Center for Physical Sciences and Technology, Akademijos 7, LT-08412 Vilnius, Lithuania; gintaras.urbelis@ftmc.lt

**Keywords:** 7-hydroxycoumarin, umbelliferone, 7-hydroxy-3,4-dihydrocoumarin, ene-reductases, alcohol dehydrogenase, *Pseudomonas mandelii*

## Abstract

Coumarins are well known secondary metabolites widely found in various plants. However, the degradation of these compounds in the environment has not been studied in detail, and, especially, the initial stages of the catabolic pathways of coumarins are not fully understood. A soil isolate *Pseudomonas mandelii* 7HK4 is able to degrade 7-hydroxycoumarin (umbelliferone) via the formation of 3-(2,4-dihydroxyphenyl)propionic acid, but the enzymes catalyzing the α-pyrone ring transformations have not been characterized. To elucidate an upper pathway of the catabolism of 7-hydroxycoumarin, 7-hydroxycoumarin-inducible genes *hcdD*, *hcdE*, *hcdF*, and *hcdG* were identified by RT-qPCR analysis. The DNA fragment encoding a putative alcohol dehydrogenase HcdE was cloned, and the recombinant protein catalyzed the NADPH-dependent reduction of 7-hydroxycoumarin both in vivo and in vitro. The reaction product was isolated and characterized as a 7-hydroxy-3,4-dihydrocoumarin based on HPLC-MS and NMR analyses. In addition, the HcdE was active towards 6,7-dihydroxycoumarin, 6-hydroxycoumarin, 6-methylcoumarin and coumarin. Thus, in contrast to the well-known fact that the ene-reductases usually participate in the reduction of the double bond, an alcohol dehydrogenase catalyzing such reaction has been identified, and, for *P. mandelii* 7HK4, 7-hydroxycoumarin degradation via a 7-hydroxy-3,4-dihydrocoumarin pathway has been proposed.

## 1. Introduction

Coumarins are the naturally occurring benzopyran-2-ones that are widespread between various plants in nature [1]. It is thought that these compounds play important fungicidal and bactericidal roles in plants. Keeping in mind the physiological significance of coumarins, there has been a strong interest in the turnover and detoxification mechanisms of those compounds in nature, including microorganisms [2]. It is known that the metabolism of coumarins starts with the reduction and hydrolysis of its lactone moiety in several bacterial strains [3,4]. According to literature, reduction of the activated C=C-bond in coumarin can be catalyzed by ene-reductases (ERs) [5]. ERs are widely used in a biocatalytic nicotinamide-dependent asymmetric reduction of the activated double bonds yielding chiral α-substituted and/or β-substituted reduction products. To date, four different classes of ERs capable of reducing a broad variety of substrates including α,β-unsaturated aldehydes, ketones, carboxylic acids and derivatives (such as esters, lactones, cyclic imides), and nitro compounds have been identified [6,7,8,9,10]. The most extensively investigated family of ERs is the FMN-containing the Old Yellow Enzyme (OYE) family of oxidoreductases [9]. Other families of ERs are the oxygen-sensitive FAD and [4Fe-4S]-containing clostridial enoate reductases (EnoR) [11], the leukotriene B4 dehydrogenase subfamily of medium chain dehydrogenases/reductases (MDR) [12] and the salutaridine/menthone reductase-like subfamily of short chain dehydrogenases/reductases (SDR) [13,14]. The last two families contain the less characterized NAD(P)H-dependent flavin-independent ERs that have been recently studied for their biocatalytic mechanisms [7,15].

Many homologous ERs can be found in bacteria. *Pseudomonas* spp. are ubiquitous in nature, known to metabolize a wide variety of environmental pollutants, and to harbor a large number of enzymes, including some that catalyze the reduction of α,β-unsaturated compounds [16]. Hence, the xenobiotic reductase A (XenA), a member of OYE family, from *Pseudomonas putida* 86 has the ability to reduce the C3-C4 double bond of 8-hydroxycoumarin, an intermediate of the degradation of quinoline [17].

In a previous study, a lower pathway of degradation of 7-hydroxycoumarin in *Pseudomonas mandelli* 7HK4 bacteria encoded by a *hcdABC* gene cluster and responsible for the degradation of 3-(2,4-dihydroxyphenyl)propionic acid has been described [18]. However, it is still unclear how 7-hydroxycoumarin is converted to 3-(2,4-dihydroxyphenyl)propionic acid in these bacteria.

Here, to elucidate the first step of catabolism of 7-hydroxycoumarin, xenobiotic reductases from *Pseudomonas mandelli* 7HK4 bacteria were tested as putative enzymes involved in a reduction of the lactone ring. Two of three xenobiotic reductases—XenA38 and XenA45—were active towards various coumarins, however, none of them was induced in the presence of 7-hydroxycoumarin. In contrast, an analysis of 7-hydroxycoumarin-inducible genes allowed the identification of a putative alcohol dehydrogenase HcdE, which could catalyze a reduction of 7-hydroxycoumarin to 7-hydroxy-3,4-dihydrocoumarin. To our best knowledge, the HcdE was the first alcohol dehydrogenase capable of the reduction of a double bound. Based on these data, a 7-hydroxy-3,4-dihydrocoumarin pathway of utilization of 7-hydroxycoumarin in *P. mandelii* 7HK4 cells was proposed.

## 2. Results

### 2.1. Screening for XenA Reductase Homologues in the Genome of P. mandelii 7HK4

**Figure 1:**
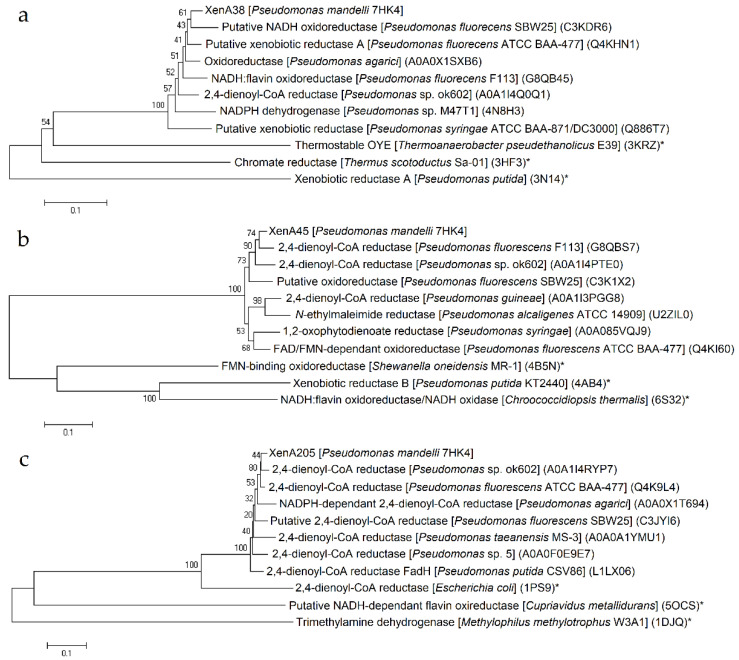
Phylogenetic analysis of XenA38 (**a**), XenA45 (**b**) and XenA205 (**c**) proteins from *Pseudomonas mandelii* 7HK4 bacteria. Neighbor joining analyses were performed on the closest homologues with or without known structures and/or function. The numbers on the nodes indicate how often (no. of times, %) the species to the right grouped together in 1000 bootstrap samples. Bars represent the number of amino acid substitutions per site. UniProt/PDB accession numbers are given in parentheses. Proteins with known structure and/or function are marked with an asterix (*).

To find out whether *P. mandelii* 7HK4 cells utilized the ERs for the catabolism of 7-hydroxycoumarin, we used the amino acid sequence of the known XenA protein to search for the homologues encoded in the genome of *P. mandelii* 7HK4 [18]. In this way, three genes denoted as *xenA38*, *xenA45* and *xenA205* encoding putative reductases with close similarity to the known XenA proteins were discovered. The products of the *xenA38*, *xenA45* and *xenA205* genes belonged to the mycofactocin system FadH/OYE oxidoreductase family. The first two proteins bore the most resemblance to a putative NADH oxidoreductase from *Pseudomonas fluorescens* SBW25 and 2,4-dienoyl-CoA reductase from *Pseudomonas fluorescens* F113, respectively (Figure 1a,b).

XenA205 protein was similar to 2,4-dienoyl-CoA reductase from *Pseudomonas* sp. ok602 (Figure 1c). All three genes were scattered throughout the genome of *P. mandelli* 7HK4 and were unlikely belonging to any putative gene clusters.

### 2.2. Characterization of Putative Xenobiotic Reductases

**Figure 2:**
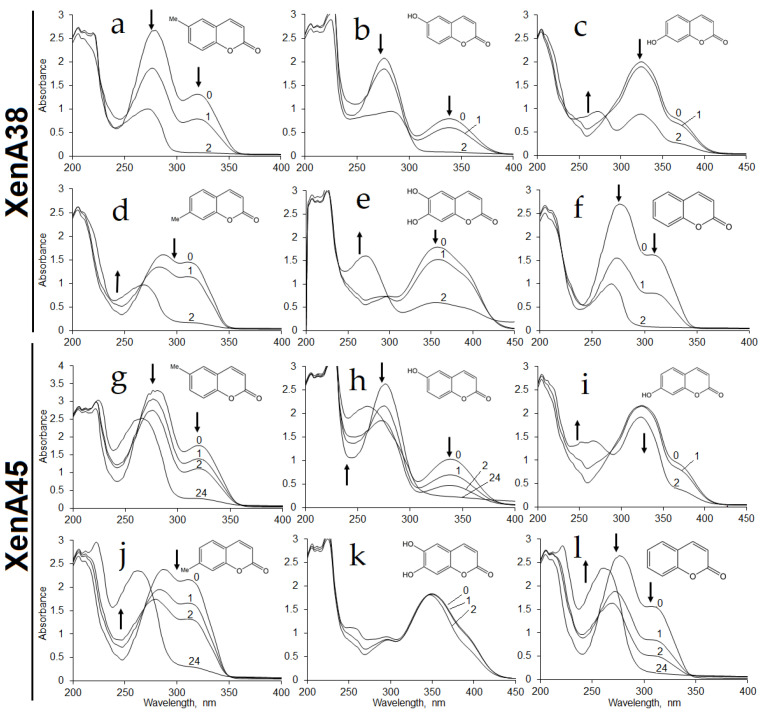
Biotransformation of 6-methylcoumarin (**a**,**g**), 6-hydroxycoumarin (**b**,**h**), 7-hydroxycoumarin (**c**,**i**), 7-methylcoumarin (**d**,**j**), 6,7-dihydroxycoumarin (**e**,**k**) and coumarin (**f**,**l**) by whole cells of *E. coli* BL21 containing *xenA38* (**a**–**f**) or *xenA45* (**g**–**l**) gene. Biotransformation was carried out in 50 mM potassium phosphate buffer pH 7.2 at 30 °C with 0.5 mM of substrate. Incubation time (hours) is shown above the curves. In several experiments (**c**,**e**,**i**,**k**), the spectra after an incubation for 24 h were omitted, due to a massive lysis of cells taking place during a prolonged incubation. Arrows indicate changes in absorbance.

*E. coli* BL21 cells transformed with the plasmid harboring *xenA38*, *xenA45* or *xenA205* gene were used for the assay of the activity of the appropriate enzyme by time course experiments. *E. coli* BL21 containing *xenA38* gene showed bioconversion of coumarin, 6-hydroxycoumarin, 6-methylcoumarin, 7-hydroxycoumarin, 7-methylcoumarin and 6,7-dihydroxycoumarin according to the changes in the UV−VIS spectra (Figure 2a–f).

Whole cells of *E. coli* BL21 containing XenA45 reductase showed similar activities against these substrates except for the reaction with 6,7-dihydroxycoumarin of which UV−VIS spectra did not change over time (Figure 2g–l). No activities were observed with *o*-coumaric acid and 2,4-dihydroxycinnamic acid. The *E. coli* cells with *xenA205* gene or without any additional genes showed no activity towards those compounds. These findings revealed that *P. mandelii* 7HK4 encode two putative xenobiotic reductases, which could utilize a number of differently substituted coumarins.

For further characterization the C-terminal His_6_-tagged XenA38 and XenA45 proteins were produced in *E. coli* BL21, and purified by affinity chromatography. Both purified enzymes migrated as ~40 kDa bands in SDS-PAGE, the solution of XenA45 enzyme was colorless contrary to the XenA38 protein that had a bright yellow color inferring that the protein contained a tightly bound flavin [19,20]. However, all attempts to measure enzymatic activity under aerobic conditions gave no results.

### 2.3. Quantitative RT-PCR Analysis of the Pseudomonas mandelii 7HK4 Transcripts Induced by Coumarins

**Figure 3:**
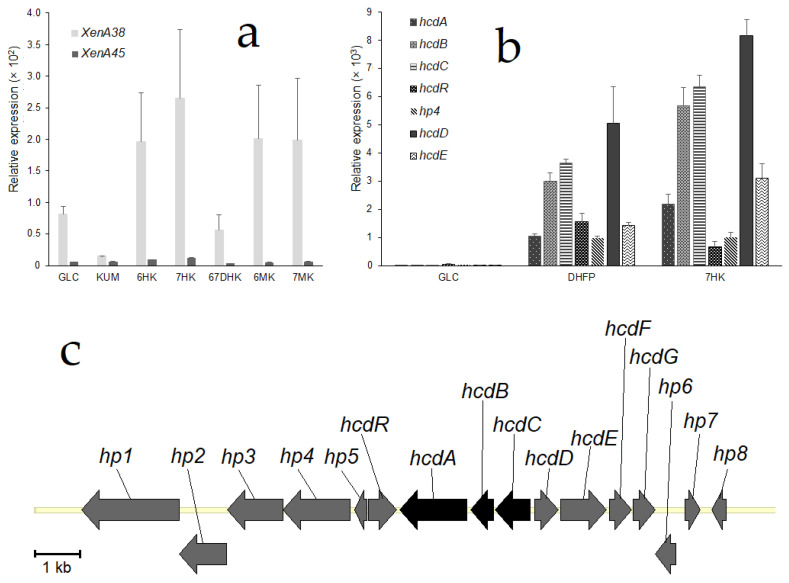
Quantitative RT-PCR analysis of *Pseudomonas mandelii* 7HK4 transcripts. Strain 7HK4 was cultivated in minimal medium supplemented with either 1 mM coumarin derivative (induced conditions) or glucose (noninduced condition) as a single source of carbon. (**a**) qPCR analysis of the transcription of *xenA38* and *xenA45* genes. (**b**) qPCR analysis of the transcription of *hcdABCR*, *hp4, hcdD* and *hcdE* genes. Primers were designed to amplify the regions of corresponding genes (Table S3). The data are presented as relative RNA amounts calculated from the threshold cycles using the threshold cycle of 16S RNA as a reference. The averages of three independent runs are presented. GLC—glucose; KUM—coumarin; 6HK—6-hydroxycoumarin; 7HK—7-hydroxycoumarin; 6MK—6-methylcoumarin; 7MK—7-methylcoumarin; 67DHK—6,7-dihydroxycoumarin; DHFP—3-(2,4-dihydroxyphenyl)propionic acid. (**c**) Organization of *hcdABC* and its adjacent genes in *P. mandelii* 7HK4 bacteria. The black arrows indicate previously described ORFs encoding HcdA, HcdB, HcdC [18] and grey arrows indicate *hcdDEFG* genes studied in this work and ORFs encoding hypothetical proteins (hp).

To investigate whether the expression of the putative reductase genes was dependent on 7-hydroxycoumarin, quantitative PCR analysis was performed. *Pseudomonas mandelii* 7HK4 cells were induced with various coumarin derivatives and total RNA was isolated as described in Materials and Methods. Results revealed that the expression of each of the *xenA38* and *xenA45* genes were not markedly induced when *P. mandelii* 7HK4 was cultivated in the presence of either 7-hydroxycoumarin, or the other coumarin derivatives (Figure 3a). Moreover, *xenA45* gene was not expressed under any tested condition, although the expression levels of the *xenA38* gene were significantly higher than its counterpart. Hence, the levels of a *xenA38* mRNA synthesis in the hydroxycoumarin-induced 7HK4 cells were compared to the ones in the glucose-grown (noninduced) cells. Thus, it was found that the transcription of the *xenA38* gene was increased two-fold in the presence of 6-hydroxycoumarin and three-fold in the presence of 7-hydroxycoumarin.

An expression of the *xenA38* gene was also compared to the expression levels of *hcdABC* genes encoding a lower part of the degradation pathway of 7-hydroxycoumarin [18]. It was found that the levels of mRNA synthesis of *hcdABC* genes were increased 1000-fold in the presence of 7-hydroxycoumarin (Figure 3b). Therefore, we concluded that *xenA38* mRNA levels are too low to be induced by coumarin derivatives and XenA38 could be considered as a constitutive enzyme that shares a broad reducing activity in the *P. mandelii* 7HK4 cells.

For further investigation, we conducted a quantitative PCR analysis of genes that are adjacent to the *hcdABC* gene cluster (Figure 3c). The results revealed that randomly chosen putative *hp4*, *hcdR*, *hcdD* and *hcdE* genes were all induced in *P. mandelii* 7HK4 cells (Figure 3b). Expression levels of *hcdR*, *hp4*, *hcdD* and *hcdE* genes were increased approximately 1000-fold, and were similar to expression levels of the *hcdABC* genes under the same conditions. Each of the *hcdABCR*, *hp4*, *hcdD* and *hcdE* genes was specifically induced when *P. mandelii* 7HK4 was cultivated in the presence of either 3-(2,4-dihydroxyphenyl)propionic acid or 7-hydroxycoumarin. In addition, a 100-fold increase of mRNA synthesis of these genes was also observed in *P. mandelii* 7HK4 cells precultivated with 7-methylcoumarin. No expression of the analyzed genes was observed in *P. mandelii* 7HK4 cells precultivated with coumarin, 6-hydroxycoumarin, 6-methylcoumarin, 6,7-dihydroxycoumarin, *o*-coumaric acid, *p*-coumaric acid, 2,4-dihydroxycinnamic acid, quinoline or isoquinoline.

### 2.4. Analysis of the Genome Locus Adjacent to 7-Hydroxycoumarin-Inducible hcd Gene Cluster

A genomic region approximately 3 kb upstream of the *hcdABC* gene cluster containing *hcdD*, *hcdE*, *hcdF* and *hcdG* genes was amplified by PCR and cloned into the pACYCDuet-1 expression vector. The recombinant HcdD, HcdE, HcdF and HcdG proteins were produced together in *E. coli* BL21 strain, and the expression was confirmed by SDS-PAGE. The whole cells of *E. coli* BL21 transformed with the pHP4-10 plasmid (Table S2) were used for the bioconversion of coumarin, 6-hydroxycoumarin, 6-methylcoumarin, 7-hydroxycoumarin, 7-methylcoumarin, 7-methoxycoumarin, 4-methyl-7-hydroxycoumarin and 6,7-dihydroxycoumarin. Time course experiments revealed the decrease of UV absorption maxima of these substrates over time. After the completion of bioconversions, there was a non-disappearing UV absorption maximum at 260–280 nm wavelengths similar to that of 3-(2,4-dihydroxyphenyl)propionic acid (see Figures S1 and S7 in the Supplementary Materials). This suggested that *E. coli* BL21 cells transformed with the pHP4-10 plasmid could catalyze the reduction and/or hydrolysis of the lactone moiety of coumarin derivatives. When activity rates were compared, a conversion of the 7-hydroxycoumarin was the fastest, and conversion of methyl- and methoxy-substituted coumarins proceeded at the lowest rate. In addition, no activity was observed with 3-hydroxycoumarin, 4-hydroxycoumarin, 7-ethoxycoumarin, *o*-coumaric acid, 2,4-dihydroxycinnamic acid, 7-hydroxyquinoline-(1*H*)-2-one, 3,4-dihydroquinoline-(1*H*)-2-one, 2-hydroxyquinoline or 3,4-dihydro-7-hydroxyquinoline-(1*H*)-2-one, which implied that one or all together studied hypothetical proteins were specific for coumarin derivatives with unsubstituted and non-hydrolyzed lactone moiety. The *E. coli* cells without the *hcdD*, *hcdE*, *hcdF* and *hcdG* genes showed no activity towards the aforementioned compounds.

**Figure 4:**
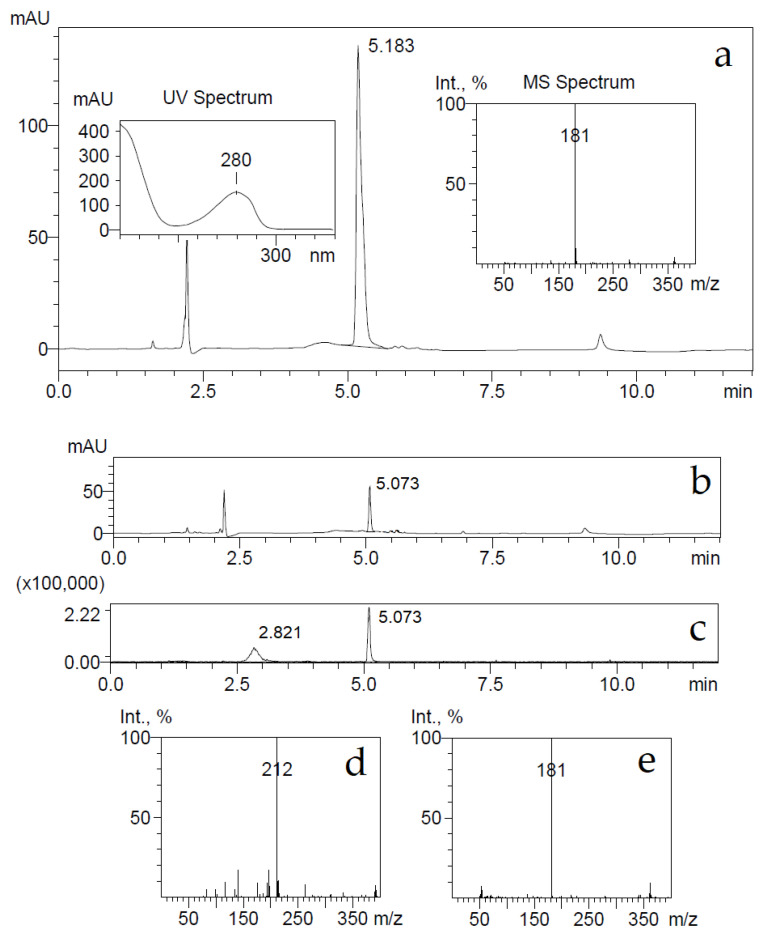
(**a**) Bioconversion of 7-hydroxycoumarin by *E. coli* BL21 bacteria harboring the pHP4-10 plasmid. Produced metabolites were analyzed by HPLC-MS. UV 280 nm trace, UV and MS spectra of metabolite with retention time 5.183 min. The negative ion [M-H]^−^ generated was at *m*/*z* 181 (3-(2,4-dihydroxyphenyl)propionic acid). (**b**) HPLC chromatogram of 7-hydroxycoumarin bioconversion by *E. coli* BL21 bacteria harboring pHP4-10, p4pmPmo and pTHPPDO plasmids. BP chromatogram (**c**) and MS spectra of metabolites with retention times 2.821 min (**d**) and 5.073 min (**e**). The negative ion [M-H]^−^ generated was at *m*/*z* 181 (3-(2,4-dihydroxyphenyl)propionic acid) and the positive ion [M+H]^+^ generated was at *m*/*z* 212 (6-(2-carboxyethyl)-4-oxo-1,4-dihydropyridine-2-carboxylic acid).

Furthermore, the recombinant HcdD, HcdE, HcdF and HcdG proteins were coexpressed with the HcdA hydroxylase and HcdB dioxygenase in *E. coli* BL21 cells, which were used for bioconversion of 7-hydroxycoumarin. The conversion products were analyzed by HPLC-MS. Ions with [M-H]^−^ masses 161 (for 7-hydroxycoumarin) (see Figure S8 in the Supplementary Materials) and 163 (for 7-hydroxy-3,4-dihydrocoumarin) were not detected showing a complete conversion of substrate and its reduced form, respectively. Moreover, the ions with [M-H]^−^ mass 181 (for 3-(2,4-dihydroxyphenyl)propionic acid) and with [M+H]^+^ mass 212 (for 6-(2-carboxyethyl)-4-oxo-1,4-dihydropyridine-2-carboxylic acid) were observable, showing the accumulation of a reduced and hydrolyzed form of the substrate that was further converted to (2*E*,4*E*)-2,4-dihydroxy-6-oxonona-2,4-dienedioic acid by HcdA and HcdB enzymes. Some part of the produced (2*E*,4*E*)-2,4-dihydroxy-6-oxonona-2,4-dienedioic acid reacted with ammonium anions that were in bioconversion broth resulting in a formation of picolinic acid (Figure 4) as described previously [18].

These results were compared to the bioconversion of 7-hydroxycoumarin by *E. coli* cells containing *hcdD*, *hcdE*, *hcdF* and *hcdG* genes only, which showed the accumulation of only one reaction product with ion [M-H]^−^ mass of 181 Da. This led to the conclusion that hypothetical HcdD, HcdE, HcdF and HcdG proteins might be used in the early stages of 7-hydroxycoumarin metabolism by *P. mandelii* 7HK4 bacteria, producing 3-(2,4-dihydroxyphenyl)propionic acid, which later could be as a substrate for proteins encoded by the *hcdABC* gene cluster.

**Figure 5:**
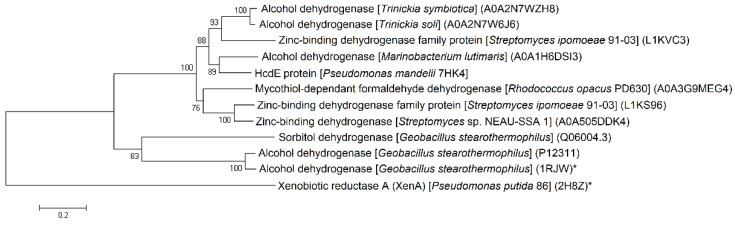
Phylogenetic tree of HcdE protein from *Pseudomonas mandelii* 7HK4 bacteria. Neighbor joining analyses were performed on the closest homologues with or without known structures and/or function. The numbers on the nodes indicate how often (no. of times, %) the species to the right grouped together in 1000 bootstrap samples. Bars represent the number of amino acid substitutions per site. UniProt/PDB accession numbers are given in parentheses. Proteins with known structure and/or function are marked with an asterix (*).

A BLAST analysis of *hcdE* and *hcdG* sequences revealed that these genes encode the putative zinc-dependent alcohol dehydrogenase (Figure 5) and NAD(P)H-dependent FMN reductase proteins, respectively. However, two other HcdD and HcdF proteins belong to less characterized groups of proteins, with similarities to *Bacillus chorismate* mutase-like (BCM-like) or cupin-like protein families, respectively.

### 2.5. Isolation and Identification of 7-Hydroxycoumarin Bioconversion Product

Previously, it was shown that *Pseudomonas* sp. 30–1 and *Aspergillus niger* ATCC 11394 utilized a putative zinc-binding and NADH-dependent oxidoreductases to reduce coumarin generating dihydrocoumarin [3,21]. Therefore, we decided to further analyze HcdE protein that has the most resemblance to alcohol dehydrogenases (Figure 5).

**Figure 6:**
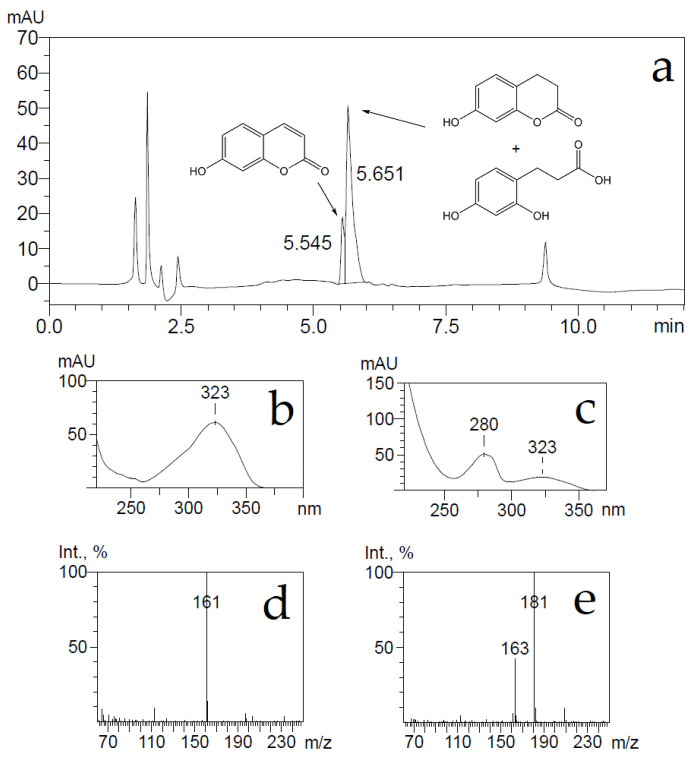
Bioconversion of 7-hydroxycoumarin by *E. coli* BL21 bacteria containing the induced *hcdE* gene. Produced metabolites were analyzed by HPLC-MS. UV 280 nm trace of metabolites (**a**). UV and MS spectra of peaks with retention times 5.545 min (**b**,**d**) and 5.651 min (**c**,**e**). The negative ions [M-H]^−^ generated were at *m*/*z* 181 (3-(2,4-dihydroxyphenyl)propionic acid), 161 (7-hydroxycoumarin) and 163 (7-hydroxy-3,4-dihydrocoumarin).

*E. coli* BL21 cells harboring the pHP7 plasmid were used for the conversion of 7-hydroxycoumarin as described in Materials and Methods. The consumption of the substrate was monitored by UV−VIS absorption spectroscopy and HPLC-MS. The UV absorption maximum changed from 340 to 270 nm, also, after conversion, a HPLC-MS analysis showed the accumulation of reaction products with ion [M-H]^−^ masses of 163 and 181 Da, and some substrate retained with ion [M-H]^−^ mass of 161 Da (Figure 6). The detected ion [M-H]^−^ masses of 181 and 161 Da implied that the bioconversion reaction was incomplete, probably due to an inhibition by the substrate, and the presumed reaction product 7-hydroxy-3,4-dihydrocoumarin might been hydrolyzed by forming 3-(2,4-dihydroxyphenyl)propionic acid.

**Figure 7:**
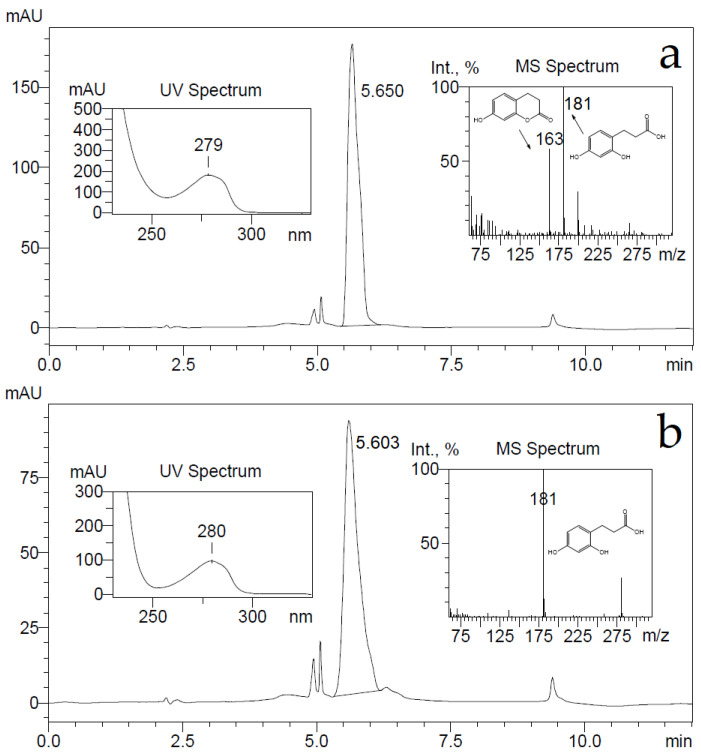
HPLC chromatograms of 7-hydroxy-3,4-dihydrocoumarin (**a**) and 3-(2,4-dihydroxyphenyl)propionic acid (**b**) at 280 nm wavelength. A sample of 2 mM of compounds was incubated in 50 mM potassium phosphate buffer (pH 7.2) for several hours and extracted by ethyl acetate. Corresponding UV and MS spectra of the main peaks are presented. The negative ions [M-H]^−^ generated are at *m*/*z* 181 (3-(2,4-dihydroxyphenyl)propionic acid) and 163 (7-hydroxy-3,4-dihydrocoumarin).

A conversion product was purified, yielding 3-(2,4-dihydroxyphenyl)propionic acid only as it was proven by HPLC-MS, ^1^H and ^13^C NMR analysis. To check whether the supposed reaction product—7-hydroxy-3,4-dihydrocoumarin—could be transformed through hydrolysis during the conversion reaction, 7-hydroxy-3,4-dihydrocoumarin was chemically synthesized as described in Materials and Methods, and the possible changes of it were investigated imitating the bioconversion conditions. Small amounts (a final concentration of 2 mM) of either 7-hydroxy-3,4-dihydrocoumarin, or 3-(2,4-dihydroxyphenyl)propionic acid were dissolved in 50 mM potassium phosphate buffer (pH 7.2) and incubated for several hours. Later, these compounds were extracted from aqueous solution with either dichloromethane, or ethyl acetate, then the solvent was removed and precipitates were dissolved in acetonitrile. Analysis of these samples by HPLC-MS showed that dichloromethane could extract 7-hydroxy-3,4-dihydrocoumarin only, on the other hand an extraction by ethyl acetate worked equally well for both analytes. Both ion [M-H]¯ masses of 163 and 181 Da were detected in the 7-hydroxy-3,4-dihydrocoumarin samples after an extraction by ethyl acetate from the aqueous solution (Figure 7).

Further, the elution time of a compound from the 7-hydroxy-3,4-dihydrocoumarin sample was the same as one from the 3-(2,4-dihydroxyphenyl)propionic acid sample. A prolonged incubation (24 h) of 7-hydroxy-3,4-dihydrocoumarin in aqueous buffer solutions led to the disappearance of the [M-H]^−^ ion mass of 163 Da in HPLC-MS analysis. This experiment confirmed that 7-hydroxy-3,4-dihydrocoumarin is prone to hydrolysis during incubation in aqueous solution and extraction by organic solvents.

Based on all experiments, it was concluded that 7-hydroxycoumarin was reduced by the HcdE enzyme resulting in the formation of 7-hydroxy-3,4-dihydrocoumarin. However, due to a strong inhibition of the HcdE enzyme by its substrate, the conversion rates were low enough that a prolonged conversion affected the reaction product 7-hydroxy-3,4-dihydrocoumarin, which underwent hydrolysis in aqueous solutions.

### 2.6. Characterization of HcdE Protein

The *hcdE* gene was expressed and the recombinant C-terminally His_6_-tagged protein was produced in *E. coli* BL21 cells. A soluble and colorless HcdE protein was purified by affinity chromatography and analyzed by SDS-PAGE. The enzyme migrated as a ~40 kDa protein band (Figure S2). The specificity of the enzyme was investigated for several cofactors, however the HcdE was able to utilize NADPH only. The addition of FAD or FMN to the reaction mixtures showed no changes in either NADH or NADPH oxidation. The optimum reaction conditions for the HcdE activity were found to be a low ionic strength phosphate-citrate buffer of pH 7.0 at room temperature (Figures S3 and S4).

**Figure 8:**
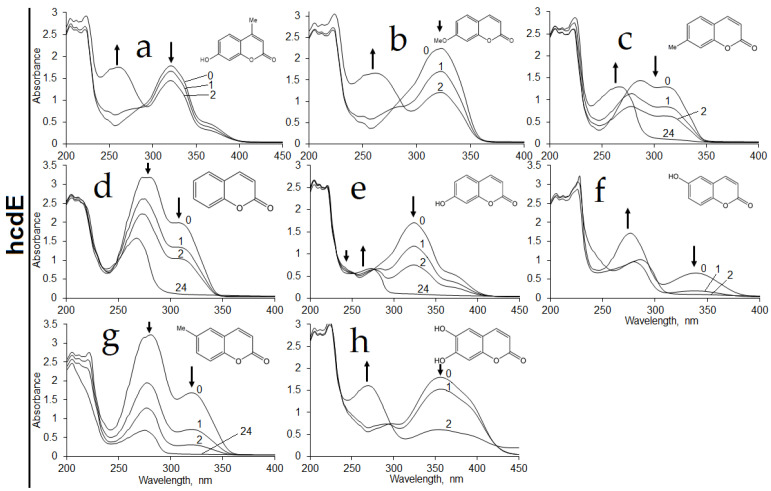
Biotransformation of 4-methyl-7-hydroxycoumarin (**a**), 7-methoxycoumarin (**b**), 7-methylcoumarin (**c**), coumarin (**d**), 7-hydroxycoumarin (**e**), 6-hydroxycoumarin (**f**), 6-methylcoumarin (**g**) and 6,7-dihydroxycoumarin (**h**) by whole cells of *E. coli* BL21 containing *hcdE* gene. Biotransformation was carried out in 50 mM potassium phosphate buffer pH 7.2 at 30 °C with 0.5 mM of substrate. Incubation time (hours) is shown above the curves. In several experiments (**a**,**b**,**h**), the spectra after an incubation for 24 h were omitted, due to a massive lysis of cells taking place during the prolonged incubation. Arrows indicate changes of absorbance.

Further, the activity of the HcdE enzyme was assayed in the presence of NADPH cofactor against various coumarin substrates. The highest enzymatic activity was recorded in the presence of 7-hydroxycoumarin. Rates lower by 1.6-, 2-, 3.4- and 17-fold were observed when 6,7-dihydroxycoumarin, 6-hydroxycoumarin, 6-methylcoumarin and coumarin were used as the substrates, respectively. The HcdE was not active towards 7-methylcoumarin, 7-methoxycoumarin, 7-ethoxycoumarin, 4-methyl-7-hydroxycoumarin, 3-hydroxycoumarin, 4-hydroxycoumarin, *trans*-cinnamic, *trans*-2,4-dihydroxycinnamic, *o*-coumaric, *m*-coumaric, *p*-coumaric and caffeic acids, cinnamyl alcohol, 7-hydroxyquinoline-(1*H*)-2-one, 3,4-dihydroquinoline-(1*H*)-2-one, 2-hydroxyquinoline, 3,4-dihydro-7-hydroxyquinoline-(1*H*)-2-one and indole (Table S1). Some data of in vitro experiments contradicted the results obtained by using the whole *E. coli* BL21 cells harboring the pHP7 plasmid. Hence, those cells showed some activities towards 7-methylcoumarin, 7-methoxycoumarin and even 4-methyl-7-hydroxycoumarin (Figure 8).

It was proposed that a strong inhibition of HcdE took place in vitro, when such effect was bypassed within *E. coli* BL21 cells by limiting substrate transportation through the cell membrane, thus maintaining a low concentration of substrate within the cells.

The initial velocities were measured for HcdE protein by varying both NADPH and 7-hydroxycoumarin concentrations to determine the kinetic properties of the HcdE enzyme. The estimation of the kinetic parameters was performed by fitting the data to the Michaelis−Menten expression of the bimolecular reaction rate (Equation (1)):

(1) $$v=\frac{[E_{0}]k_{bim}k_{NADPH}\left[NADPH\right]\left[7HK\right]}{k_{NADPH}\left[NADPH\right]+k_{bim}\left[7HK\right]\left(K_{m}^{NADPH}+\left[NADPH\right]+K_{i}\left[NADPH\right]\left[7HK\right]\right)}$$

where [NADPH] was the concentration of NADPH; [7HK] was the concentration of 7-hydroxycoumarin; *v* was the observed rate; *k_bim_* was the bimolecular rate constant of 7-hydroxycoumarin reduction, expressed as $k_{7HK}/K_{m}^{7HK}$ (where $K_{m}^{7HK}=\left(k_{m2}+k_{7HK}\right)/k_{2}$); *k_NADPH_* was the rate constant of NADPH oxidation; $K_{m}^{NADPH}$ was the *K*_m_ for NADPH at saturating 7-hydroxycoumarin levels (where $K_{m}^{NADPH}=\left(k_{m1}+k_{NADPH}\right)/k_{1}$); and *K*_i_ was the inhibition constant of 7-hydroxycoumarin. The action of 7-hydroxycoumarin reduction and NADPH oxidation was represented by the proposed reaction schemes (Equations (2)–(4)).

(2) $$E_{0}\;+\mathrm{NADPH}\;\overset{k_{1}/k_{m1}}{\Leftrightarrow }\;E_{1}\;\overset{k_{NADPH}}{\to }\;E_{2}$$

(3) $$E_{2}\;+7\mathrm{HK}\;\overset{k_{2}/k_{m2}}{\Leftrightarrow }\;E_{3}\;\overset{k_{7HK}}{\to }\;E_{0}\;+{\mathrm{NADP}}^{+}\;+7{\mathrm{HKH}}_{2}$$

(4) $$E_{1}\;+7\mathrm{HK}\;\overset{k_{a}/k_{d}}{\Leftrightarrow }\;E_{i}$$

The reductive half-reaction sequence was modeled as shown in Equation (2), where E_0_ was an oxidized form of HcdE, E_1_ was the hcdE_ox_-NADPH charge-transfer complex, and E_2_ was a reduced form of HcdE bound to NADP^+^. The oxidative half-reaction sequence was modeled as shown in Equation (3), where 7HK was 7-hydroxycoumarin, E_3_ was hcdE_red_-7HK charge-transfer complex and bound NADP^+^, and 7HKH_2_ was 7-hydroxy-3,4-dihydrocoumarin.

It was also observed that 7-hydroxycoumarin acted as a strong inhibitor of the HcdE enzyme at higher concentrations (see Figure S5). We proposed that inhibition occurred when 7-hydroxycoumarin bound to the hcdE_ox_-NADPH charge-transfer complex (E_1_) rather than the oxidized HcdE enzyme form (E_0_) (Equation (4)). Therefore, 7-hydroxycoumarin was likely a pseudo-competitive inhibitor characterized by the inhibition constant *K_i_* (expressed as $k_{d}/k_{a}$) that was equal to 22 mM for 7-hydroxycoumarin. The determined *k_bim_* value of reduction of 7-hydroxycoumarin by HcdE was equal to 1490 mM^−1^s^−1^. Additionally, a rate constant (*k_cat_*) of 27 s^−1^ and a $K_{m}^{NADPH}$ of 20 μM have been determined for NADPH.

## 3. Discussion

Thus far, only a few studies have been published discussing the reduction of coumarins in microorganisms [3,21]. To date, it was known that coumarin could be reduced at the double bond in the α-pyrone ring by coumarin reductase and NADH as the first step resulting in dihydrocoumarin, followed by hydrolysis between the oxygen and carbonyl carbon atoms of the ring [3,21]. On the other hand, the initial attack on coumarin could also be hydrolysis to *o*-coumaric acid, only then followed by reduction to melilotic acid [4]. Some enzymes having coumarin reducing activity have been biochemically characterized [3,17], however scarce genetic data or enzyme mechanisms have been presented. In the present study, we discovered a 7-hydroxycoumarin-inducible alcohol dehydrogenase HcdE responsible for reduction of the α-pyrone ring of 7-hydroxycoumarin in *P. mandelii* 7HK4 bacteria.

The HcdE protein is expressed in *P. mandelii* 7HK4 cells when grown with 7-hydroxycoumarin as the sole source of carbon and energy. HcdE catalyzes the NADPH-dependent reduction of several coumarin derivatives including coumarin itself, 6-methylcoumarin, 6-hydroxycoumarin, 7-hydroxycoumarin and 6,7-dihydroxycoumarin. HcdE appears to have a high specificity for coumarin derivatives with unsubstituted and non-hydrolyzed lactone moiety, thus other structurally related substrates, such as quinolin-2(1*H*)-one or 7-hydroxyquinolin-2(1*H*)-one are not used by the HcdE enzyme. There is also one discrepancy between conversions with whole cells and pure enzyme in this research. Whole cells of *E. coli* BL21 harboring *hcdE* gene are able to reduce low levels of 7-methylcoumarin, 7-methoxycoumarin and 4-methyl-7-hydroxycoumarin, while the purified HcdE enzyme alone is not. Although the results are not conclusive, we propose that the whole cells limit substrate transportation through the cell membrane maintaining a noninhibitive concentration of substrate within cells. We showed that the HcdE dehydrogenase is inhibited by its substrate 7-hydroxycoumarin at higher concentrations with the inhibition constant *K*_i_ that is equal to 22 mM for 7-hydroxycoumarin. The inhibition occurs when 7-hydroxycoumarin binds to the HcdE_ox_-NADPH charge-transfer complex (E_1_), thus reducing the NADPH oxidation rate.

HcdE dehydrogenase is, to our best knowledge, the first ene-reductase of the MDR family involved in the in vivo degradation of coumarin compounds. Only one functionally related enzyme, such as XenA reductase from *Pseudomonas putida* 86 is known [17]. XenA reductase has well-described primary and secondary structures, yet it belongs to another family of ERs—OYEs, catalyzing FMN and NAD(P)H dependent reduction of coumarin and 8-hydroxycoumarin. The determined *k_bim_* value of reduction of 7-hydroxycoumarin by HcdE dehydrogenase was equal to 1490 mM^−1^s^−1^. Additionally, a rate constant (*k_cat_*) of 27 s^−1^ has been determined for NADPH oxidation by hcdE dehydrogenase, which is almost 10-fold lower compared to the rate constant of 215 s^−1^ for NADPH oxidation by XenA reductase from *Pseudomonas putida* 86 [10,22]. The difference in NADPH oxidation between two functionally similar hcdE and XenA reductases could be due to observable competitive inhibition of HcdE enzyme by its substrate while XenA reductase is not inhibited.

**Figure 9:**
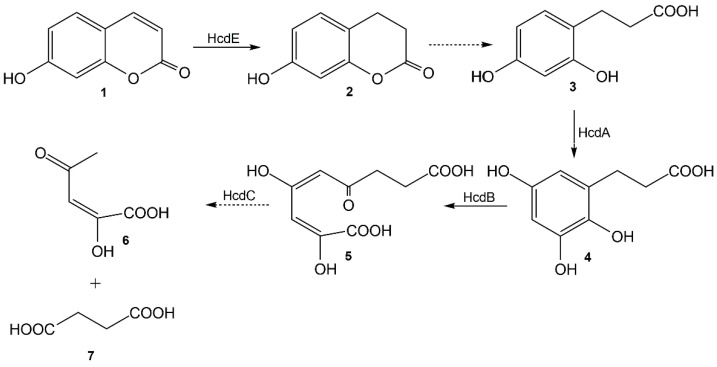
The proposed catabolic pathway of 7-hydroxycoumarin in *Pseudomonas* sp. 7HK4 bacteria. **1**—7-hydroxycoumarin; **2**—7-hydroxy-3,4-dihydrocoumarin; **3**—3-(2,4-dihydroxyphenyl)propionic acid; **4**—3-(2,3,5-trihydroxyphenyl)propionic acid; **5**—(2*E*,4*E*)-2,4-dihydroxy-6-oxonona-2,4-dienedioic acid; **6**—(*E*)-2-hydroxy-4-oxopent-2-enoic acid; **7**—succinic acid; HcdA—3-(2,4-dihydroxyphenyl)propionic acid 1-monooxygenase; HcdB—3-(2,3,5-trihydroxyphenyl)propionic acid 1,2-dioxygenase; HcdC—putative (2*E*,4*E*)-2,4-dihydroxy-6-oxonona-2,4-dienedioic acid hydrolase; HcdE—7-hydroxycoumarin reductase. The dashed arrows indicate the hypothetical reactions.

The bioconversion of 7-hydroxycoumarin was completed in vivo by the HcdE protein, and the reduced product was confirmed by HPLC-MS and NMR analysis as well as by chemical synthesis to be 7-hydroxy-3,4-dihydrocoumarin. However due to a strong inhibition of the HcdE enzyme by its substrate the conversion rates were low enough that the prolonged conversion time affected the reaction product 7-hydroxy-3,4-dihydrocoumarin. The latter compound is a lactone (a cyclic ester) that is much more reactive than its acyclic analogs. Additionally, it has a phenyl ring attached and the fact that it is a phenolic ester, 7-hydroxy-3,4-dihydrocoumarin is significantly more reactive against hydroxyl anions. Hence, a hydrolysis in aqueous solutions takes place, resulting in the formation of 3-(2,4-dihydroxyphenyl)propionic acid. Therefore, this work in combination with the previously published data [18] allow an elucidation of a pathway of catabolism of 7-hydroxycoumarin in *Pseudomonas* sp. 7HK4 bacteria (Figure 9).

During the analysis of the *P. mandelii* 7HK4 genome sequences adjacent to the *hcdE* gene it was also expected to detect genes encoding hydrolase-like enzymes that could be responsible for the hydrolysis of 7-hydroxy-3,4-dihydrocoumarin. Transcriptional analysis revealed that like *hcdE*, the *hcdD* gene was also induced in *P. mandelii* 7HK4 cells pregrown with 7-hydroxycoumarin. In addition, *hcdD* and *hcdE* together with *hcdF* and *hcdG* genes are arranged on the same DNA strand, and are separated by short intergenic regions, suggesting that these genes are organized into an operon. The HcdD protein is similar to *Bacillus* chorismate mutase-like (BCM-like) family proteins, and is in agreement with the first representatives of Pfam family (formerly DUF1185) [23]. We propose that this protein can stimulate a 7-hydroxy-3,4-dihydrocoumarin formation similarly to the homologous protein TgnF, which stimulates TgnE-dependent succinic acid semialdehyde oxidation in *Acinetobacter baylyi* ADP1 bacteria [24]. However, all attempts to observe such an effect in the case of HcdD have been unsuccessful. Further, HcdF is a cupin-like protein, which can be classified as a member of the RmlC-like cupins superfamily. It comprises families with members displaying diverse functions ranging from enzymatic activities like dioxygenases, hydrolases and epimerases to nonenzymatic functions [25,26,27]. We believe that HcdF might act as a 7-hydroxy-3,4-dihydrocoumarin hydrolase or as an enzyme catalyzing removal of a methoxy group from the 7-*O*-methylated 7-hydroxycoumarin, however further investigation is needed to elucidate its exact function in *P. mandelii* 7HK4 cells. The last protein encoded by the *hcdDEFG* gene cluster—HcdG—belongs to the NAD(P)H-dependent FMN reductase superfamily, members of which are widely involved in the electron-transfer reactions required for the metabolism of various compounds in bacteria [28,29,30].

Finally, an in silico analysis of the *P. mandelii* 7HK4 genome provided evidence that 7HK4 cells harbor also other ER enzymes capable of utilizing various coumarin derivatives. Two putative xenobiotic reductases XenA38 and XenA45 from 7HK4 strain were produced in *E. coli* bacteria, and their activities have been determined in vivo. Coumarin, 6-hydroxycoumarin, 6-methylcoumarin, 7-hydroxycoumarin, 7-methylcoumarin and 6,7-dihydroxycoumarin are reduced by the XenA38 and XenA45 reductases. However, an analysis of mRNA synthesis shows that the production of XenA38 and XenA45 proteins are not induced by any coumarin in *P. mandelii* 7HK4 cells, although XenA38 enzyme has higher expression levels in 7HK4 cells grown in the absence of coumarins. An elucidation of a role of such enzymes as XenA38 with the constitutive expression and a broad substrate specificity needs additional studies.

In summary, in addition to previously reported *hcdABC* gene cluster that is responsible for the degradation of 3-(2,4-dihydroxyphenyl)propionic acid [18] here we report the fundamentally new insights into a 7-hydroxycoumarin catabolic pathway in *Pseudomonas mandelii* 7HK4 bacteria. A new gene encoding an enzyme responsible for the reduction of 7-hydroxycoumarin has been isolated and identified. Our results show that the reduction of 7-hydroxycoumarin in *Pseudomonas mandelii* 7HK4 involves a similar approach such as in the previously characterized coumarin catabolic routes in *Pseudomonas* sp. 30–1 and *Aspergillus niger* ATCC 11394 [3,21]. However, it has been shown that *Pseudomonas mandelii* 7HK4 bacteria employ a unique NADPH-dependent alcohol dehydrogenase HcdE for the reduction of the C-3/C-4 double bond of 7-hydroxycoumarin lactone moiety forming 7-hydroxy-3,4-dihydrocoumarin. The HcdE dehydrogenase described in this paper has a significantly distant sequence homology (identity of 14.1%, only) from the previously characterized enzymes implicated in the degradation of structurally similar substrate, for example, such as 8-hydroxycoumarin in *Pseudomonas putida* 86 [17].

Thus, the HcdE provides not only a fundamentally new insight into the degradation of hydroxycoumarins by soil microorganisms, but also has a potential in alkene hydrogenation that is one of the most widely used industrial reactions producing fine chemicals, pharmaceuticals and agrochemical intermediates [9,31,32]. Further studies are required to explore various possibilities of biocatalytic and chemo-enzymatic approaches using the HcdE dehydrogenase.

## 4. Materials and Methods

### 4.1. Bioconversions with Whole Cells

*E. coli* BL21 (DE3) bacteria containing recombinant genes were grown aerobically in 20 mL of LB medium at 30 °C overnight. High density bacterial culture was centrifuged and re-suspended in 30 mL of minimal C-750501 medium [33,34], in which the synthesis of proteins was induced with 1 mM of IPTG after 1.5 h incubation at 20 °C. Incubation at 20 °C was continued for another 24–48 h. *E. coli* cells were sedimented by centrifugation (3220× *g*, 15 min). The collected cells were washed twice with 15 mL of 0.9% NaCl solution. For whole cell conversion experiments, cells from 20 mL of culture were resuspended in 1 mL of 50 mM potassium phosphate buffer (pH 7.2). All small-scale bioconversions with whole cells were made in 50 mM potassium phosphate buffer, pH 7.2, which contained 0.5–1 mM of the substrate. The reaction mixtures were kept in thermoblock at 30 °C and 500 rpm. Bioconversion mixtures were centrifuged for 2 min at 10,000× *g* and 100 µL of the supernatant were analyzed by absorption measurements in the UV−VIS range (200–450 nm). Measurements were repeated to record the changes in the absorption intensity over time. All measurements were performed with PowerWave XS microplate reader (BioTek Instruments, Inc., Highland Park, IL, USA).

### 4.2. Quantitative RT-PCR

*P. mandelii* 7HK4 was cultivated overnight in minimal medium containing 0.05% of glucose as the sole carbon source. Then cells were sedimented by centrifugation (3220× *g*, 10 min) and resuspended in 50 mM potassium phosphate buffer, pH 7.2. *P. mandelii* 7HK4 cells were supplemented with 1 mM of various coumarin derivatives and incubated for additional 3 h at 30 °C with shaking. Total RNA was isolated using a RiboPure Bacteria RNA Purification Kit (Thermo Fisher Scientific, Vilnius, Lithuania). cDNA synthesis was performed using a High-Capacity cDNA Reverse Transcription Kit (Thermo Fisher Scientific, Vilnius, Lithuania) with 340 ng input of total RNA per sample. Quantitative-PCR (qPCR) amplification was performed using a Fast SYBR Green Master Mix (Thermo Fisher Scientific) on 7500 Fast Real-time PCR system (Thermo Fisher Scientific, Vilnius, Lithuania). qPCR was conducted in 20 µL of reaction mixture containing 10 µL of Fast SYBR Green Master Mix, 500 nM of each primer (Table S3), and 2 µL of the cDNA sample. For quantitative analysis, fluorescence data were recorded after the annealing step. All experiments were carried out in triplicate. To verify the absence of DNA in the total RNA samples, the qPCR was performed directly for RNA samples. The threshold cycle (CT) (threshold value, 5% of amplification curve plateau) values were obtained using 7500 Fast Real-time PCR Software v2.0 (Thermo Fisher Scientific, Vilnius, Lithuania).

### 4.3. Protein Purification

Proteins were purified with Äkta purifier 900 chromatography systems (GE Healthcare, Uppsala, Sweden). Cell-free extracts were applied to Ni^2+^ Chelating HiTrap HP column (GE Healthcare, Uppsala, Sweden) equilibrated with 50 mM potassium phosphate buffer, pH 7.0, at 1.0 mL/min. The column was washed with at least 3 volumes of the same buffer. Then the bound proteins were eluted with 0.5 M imidazole in 50 mM potassium phosphate buffer, pH 7.0. The fractions containing the purified enzyme applied to HiTrap Desalting column with Sephadex G-25 resin (GE Healthcare, Uppsala, Sweden) and eluted with 50 mM potassium phosphate buffer, pH 7.0. Proteins were analyzed by SDS-PAGE according Laemmli [35]. Protein concentration was determined by Lowry method [36].

### 4.4. Enzyme Assay

Reducing activity of HcdE protein was measured spectrophotometrically by monitoring absorption changes of bimolecular reaction at 340 nm wavelength due to the oxidation of NADPH and reduction of various coumarin derivatives (*ε*_340_ = 5580 M^−1^cm^−1^ (for 7-hydroxycoumarin), *ε*_340_ = 4780 M^−1^cm^−1^ (for 6-hydroxycoumarin), *ε*_340_ = 4440 M^−1^cm^−1^ (for 6-methylcoumarin), *ε*_340_ = 8500 M^−1^cm^−1^ (for 6,7-dihydroxycoumarin), *ε*_340_ = 3690 M^−1^cm^−1^ (for coumarin)) after the addition of the enzyme. The activity measurements were made with cell-free extracts or the purified protein. The standard measurements of the enzyme activity were carried out at room temperature in 0.8 mL of reaction mixture, containing 50 mM potassium phosphate buffer (pH 7.0), 160 µM NADPH, and 60 µM aromatic substrate.

Kinetic characterization of HcdE protein was performed by monitoring absorption changes of reaction mixture at 365 nm wavelength due to the oxidation of NADPH only (*ε*_365_ = 3500 M^−1^cm^−1^), after the addition of the enzyme. The measurements of the enzyme activity were carried out at room temperature in 0.8 mL of reaction mixture, containing 50 mM potassium phosphate buffer (pH 7.0), 5–200 µM NADPH, 5–150 µM 7-hydroxycoumarin, and 99 nM HcdE protein. Each measurement was performed three times. Kinetic data were analyzed using Wolfram Mathematica software (Wolfram Research, Inc., Oxfordshire, UK).

### 4.5. In Vivo Bioconversion of 7-Hydroxycoumarin and Purification of Bioconversion Products

*E. coli* BL21 (DE3) cells, containing pHP7 plasmid, were grown in 6 × 200 mL of LB medium at 30 °C and 180 rpm overnight. High density bacterial cultures were supplied with 0.5 mM of IPTG for induction of protein synthesis, and incubated at 20 °C and 180 rpm. After 48 h of induction cells were centrifuged and resuspended in respective volumes of 50 mM potassium phosphate buffer (pH 7.2). Then, 7-hydroxycoumarin was added to the final concentration of 0.3 mM. Bioconversion mixture was incubated at 30 °C with shaking overnight. Cells were removed by centrifugation for 40 min at 3220× *g* and the supernatants were used for product purification.

Bioconversion product was purified as described in [37] with minor changes. The biotransformation broth was treated with concentrated HCl aq. in order to bring the pH between 4 and 5. The solution was saturated with NaCl and extracted with CH_2_Cl_2_ (4 × 100 mL). The combined organic phases were dried (Na_2_SO_4_) and concentrated under reduced pressure resulting in 7-hydroxycoumarin residues (MS (ESI+): *m*/*z* 161 [M-H]^−^). Ethyl acetate (100 mL) was then added to the aqueous solution and the mixture was filtered on a celite pad. The phases were separated and the aqueous phase was extracted with further solvent. The combined organic phases were dried (Na_2_SO_4_) and concentrated under reduced pressure. The obtained pale-yellow precipitates consisted of 3-(2,4-dihydroxyphenyl)propionic acid. MS (ESI+): *m*/*z* 181 [M-H]^−^; ^1^H NMR (DMSO-*d*_6_, 400 MHz): δ 11.97 (s, 1H), 9.14 (s, 1H), 8.96 (s, 1H), 6.80 (d, *J* = 8.2 Hz, 1H), 6.26 (d, *J* = 2.4 Hz, 1H), 6.11 (dd, *J* = 8.1, 2.4 Hz, 1H), 2.63 (dd, *J* = 8.6, 6.9 Hz, 2H), 2.40 (dd, *J* = 8.5, 6.9 Hz, 2H); ^13^C NMR (DMSO-*d*_6_, 100 MHz): δ 174.73, 157.00, 156.23, 130.41, 117.79, 106.33, 102.84, 34.63, 25.37. Given NMR spectra complies with known spectra of 3-(2,4-dihydroxyphenyl)propionic acid standard (see section NMR spectra of 3-(2,4-dihydroxyphenyl)propionic acid standard in Supplementary Material). The yield of 3-(2,4-dihydroxyphenyl)propionic acid from 59 mg of starting material was 50 mg (75.7% of the theoretical yield).

### 4.6. Organic Synthesis of 7-Hydroxy-3,4-Dihydrocoumarin

The analytical sample of 7-hydroxy-3,4-dihydrocoumarin was synthesized in good yield by thermal cyclization of 3-(2,4-dihydroxyphenyl)propionic acid [38]. In short, 3-(2,4-dihydroxyphenyl)propionic acid (182 mg, 1 mmol) was heated in an oven at 135 °C for 3 h. Resulting brown colored melt was cooled to room temperature and dissolved in a hot toluene. The solution was treated with silica, filtered and the solvent was removed under vacuum. The residue then was crystallized from a small amount of toluene. The product was filtered and vacuum dried; Yield: 112 mg (68%). MS (ESI+): *m*/*z* 163 [M-H]^−^ (see Figure S6). ^1^H NMR (400 MHz, DMSO-*d*_6_): δ 9.61 (s, 1H), 7.05 (d, *J* = 8.2 Hz, 1H), 6.52 (dd, *J* = 8.2, 2.4 Hz, 1H), 6.43 (d, *J* = 2.4 Hz, 1H), 2.86–2.82 (m, 2H), 2.74–2.70 (m, 2H). ^13^C NMR (100 MHz, DMSO-*d*_6_): δ 168.5, 157.1, 152.3, 128.7, 113.2, 111.2, 103.2, 29.0, 22.1.

### 4.7. Other Analytical Methods

For the HPLC-MS analysis, most of the analytes were dissolved in acetonitrile, otherwise the aqueous samples were mixed with an equal part of acetonitrile and centrifuged before the analysis. High-performance liquid chromatography and mass spectrometry (HPLC-MS) were carried out using the system consisting of the CBM-20 control unit, two LC-2020AD pumps, SIL-30AC auto sampler and CTO-20AC column thermostat, using the SPD-M20A detector and LCMS-2020 mass spectrometer with ESI source (Shimadzu, Japan).

Chromatographic fractionation was conducted using YMC-Pack Pro C_18_ column, 150 × 3 mm (YMC) at 40 °C, with water and acetonitrile gradient from 5% to 95%.

Mass spectra were recorded from *m*/*z* 10 up to 500 *m*/*z* at 350 °C and ±4500 V using N_2_. Mass spectrometry analysis was carried out using both the positive and negative ionization modes. The data were analyzed using LabSolutions LC/MS software (Shimadzu, Japan).

^1^H NMR and ^13^C NMR spectra were recorded in DMSO-*d*_6_ on Avance III 400 NMR spectrometer, at 400 MHz for ^1^H and 100 MHz for ^13^C, chemical shifts are reported in ppm relative to solvent resonance signal as an internal standard (^1^H NMR: δ (DMSO-*d*_6_) = 2.50 ppm; ^13^C NMR: δ (DMSO-*d*_6_) = 39.52 ppm).

### 4.8. Accession Numbers

Accession numbers for the sequences of HcdE, XenA38 and XenA45 genes are MW310254, MW310255, MW310256, respectively. The whole fragment of *P. mandelii* 7HK4 genome containing *hcd* genes (Figure 3c) could be found by the accession number MW310253.

## Data Availability

Data are contained within the article or supplementary material.

## Supplementary Materials

Supplementary materials can be found at https://www.mdpi.com/1422-0067/22/4/1552/s1. Table S1: Materials and reagents used in the studies, Table S2: Plasmids used in the studies, Table S3: The list of primers used in this study, Figure S1: UV−Vis spectrum of 3-(2,4-dihydroxyphenyl)propionic acid, Figure S2: SDS-PAGE of His_6_-tagged HcdE protein purified by affinity chromatography, Figure S3: Activity of HcdE protein in different buffer systems and pH, Figure S4: Activity of HcdE protein in buffers of different ionic strength, Figure S5: A 3D plot of HcdE enzyme kinetic titration data their fit, Figure S6: HPLC chromatogram of 7-hydroxy-3,4-dihydrocoumarin, Figure S7: HPLC chromatogram of 3-(2,4-dihydroxyphenyl)propionic acid, Figure S8: HPLC chromatogram of 7-hydroxycoumarin.
