# Supplementary material for: The First Step of Biodegradation of 7-Hydroxycoumarin in Pseudomonas mandelii 7HK4 Depends on an Alcohol Dehydrogenase-Type Enzyme
Source: Int J Mol Sci. 2021 Feb 4;22(4):1552. doi: 10.3390/ijms22041552 (PMC7913881; doi:10.3390/ijms22041552)
Supplement: Supplementary file 1 [file ijms-22-01552-s001.pdf]

Supplementary Information

The first step of biodegradation of 7-hydroxycoumarin in *Pseudomonas mandelii* 7HK4 depends on an alcohol dehydrogenase-type enzyme

Arūnas Krikštaponis [1], Gintaras Urbelis [2] and Rolandas Meškys [1]

[1] Department of Molecular Microbiology and Biotechnology, Institute of Biochemistry, Life Sciences Center, Vilnius University, Sauletekio al. 7, Vilnius LT-10257, Lithuania
[2] Department of Organic Chemistry, Center for Physical Sciences and Technology, Akademijos 7, Vilnius LT-08412, Lithuania

Table S1. Materials and reagents used in the studies.

**Chemicals and reagents**	**Source**
7-Hydroxycoumarin, ethyl acetate, methanol	Merk
Ampicillin, streptomycin, 3-(2-hydroxyphenyl)-2-propenoic acid (*o*-coumaric acid), 3-(4-hydroxyphenyl)-2-propenoic acid (*p*-coumaric acid), coumarin, cinnamyl alcohol, 3-(2,4-dihydroxyphenyl)propionic acid, caffeic acid	Fluka
3-Hydroxycoumarin, 4-hydroxycoumarin, 7-methylcoumarin, kanamycin sulfate, *trans*-2,4-dihydroxycinnamic acid, *trans*-cinnamic acid, indoline, indole, 6,7-dihydroxycoumarin, quinoline, isoquinoline, 7-methoxycoumarin, 4-methyl-7-hydroxycoumarin, 7-ethoxycoumarin, 3,4-dihydroquinoline-(1H)-2-one, 2-hydroxyquinoline, 3,4-dihydro-7-hydroxyquinoline-(1H)-2-one	Sigma-Aldrich
7-hydroxyquinoline-(1H)-2-one	Biosynth Carbosynth
3-(2,4-dihydroxyphenyl)propionic acid, 3,4-dihydro-7-hydroxycoumarin	This study
Succinic acid, glucose	Labochema
Agar, Brain Heart Infusion Broth (Bhi), Lysogeny broth (LB)	Oxoid
Restriction endonucleases, Phusion High-Fidelity PCR Master Mix with HF Buffer, Isopropyl β-D-1-thiogalactopyranoside (IPTG), PageRuler Prestained Protein Ladder, RiboPure™ Bacteria RNA Purification Kit, High-Capacity cDNA Reverse Transcription Kit, Fast SYBR™ Green Master Mix	Thermo Fischer Scientific Baltics
C_{18} Reverse-Phase column (12 g)	Grace

Table S2. Plasmids used in the studies.

**Plasmids**	**Properties**	**Source**
pET21b(+)	Amp^R, *lacI*, P_{T7lac}, 5442 bp	Novagen, Germany
pET28b(+)	Kan^R, *lacI*, P_{T7lac}, 5368 bp	Novagen, Germany
pCDFDuet-1	Sm^R, *lacI*, P_{T7lac}, 3781 bp	Novagen, Germany
pACYCDuet-1	Cm^R, *lacI*, P_{T7lac}, 4008 bp	Novagen, Germany
p4XenA38	The *xenA38* gene is cloned into pET21b(+), *Nde*I and *Xho*I restriction sites	This study

p4XenA45	The *xenA45* gene is cloned into pET21b(+), *Nde*I and *Xho*I restriction sites	This study
p4XenA205	The *xenA205* gene is cloned into pET21b(+), *Nde*I and *Xho*I restriction sites	This study
pTHPPDO	The *hcdB* gene is cloned into pET28b(+), *Nco*I and *Hind*III restriction sites	Previous study [1]
p4pmPmo	The *hcdA* gene is cloned into pET21b(+), *Nde*I and *Xho*I restriction sites	Previous study (1)
pHP4-10	The 3kb region containing *hcdD*, *hcdE*, *hcdF* and *hcdG* genes is cloned into pACYCDuet-1, *Nde*I and *Xho*I restriction sites	This study
pHP7	The *hcdE* gene is cloned into pET21b(+), *Nde*I and *Xho*I restriction sites	This study

Table S3. The list of primers used in this study.

**Primers**	**Primer sequence, 5'-3'**	**Features, target**	**Source**
4XenA38Nde_F	gtaattccatatgagtctgctgcttgaacc	*Nde*I restriction site, *xenA38* gene	This study
4XenA38tXho_R	aaattctcgagtcaatcacgcaaatccgactc	*Xho*I restriction site, STOP codon, *xenA38* gene	This study
4XenA45Nde_F	gtaattccatatggagcgtcccatgcccgt	*Nde*I restriction site, *xenA45* gene	This study
4XenA45tXho_R	aaattctcgagttaaaccagggtcatcaacgcct	*Xho*I restriction site, STOP codon, *xenA45* gene	This study
4XenA205Nde_F	gtaattccatatggagttaccgatggccgc	*Nde*I restriction site, *xenA205* gene	This study
4XenA205tXho_R	aaattctcgagttacaactcagcagccaaccg	*Xho*I restriction site, STOP codon, *xenA205* gene	This study
4hp4-16Nde_F	gtaattccatatgatgtcgcttgtgatggtt	*Nde*I restriction site, *hcdD* gene	This study
4hp7-16Nde_F	gtaattccatatgttggagaacatcatgac	*Nde*I restriction site, *hcdE* gene	This study
4hp7-16Xho_R	aaattctcgagtcaaggacagatgacgtagtt	*Xho*I restriction site, STOP codon, *hcdE* gene	This study
4hp10-16Xho_R	aaattctcgagttaagccaagcgcctgattt	*Xho*I restriction site, STOP codon, *hcdG* gene	This study
4XenA38qPCR_F	taaacatggcagcgtcaaac	*xenA38* gene, primer for qPCR	This study
4XenA38qPCR_R	gtttagccgaagcgacaaag	*xenA38* gene, primer for qPCR	This study
4XenA45qPCR_F	cccagcaagatatccaggac	*xenA45* gene, primer for qPCR	This study
4XenA45qPCR_R	ctgccttcccagaagaactg	*xenA45* gene, primer for qPCR	This study

4XenA205qPCR_F	aagcagatcagcgatttcgt	*xenA205* gene, primer for qPCR	This study
4XenA205qPCR_R	ggcgaggaactggttaatga	*xenA205* gene, primer for qPCR	This study
4hcdAqPCR_F	ggttttatcggtcgattcca	*hcdA* gene, primer for qPCR	This study
4hcdAqPCR_R	ctccatcttcaagcccagtc	*hcdA* gene, primer for qPCR	This study
4hcdBqPCR_F	aagtcatccagcgagtccag	*hcdB* gene, primer for qPCR	This study
4hcdBqPCR_R	atcggctgtttttcatgacc	*hcdB* gene, primer for qPCR	This study
4hcdCqPCR_F	cgtcgttgaagcaggtgtaa	*hcdC* gene, primer for qPCR	This study
4hcdCqPCR_R	aggcctcaggtatcagagca	*hcdC* gene, primer for qPCR	This study
4hcdRqPCR_F	gaagtcgcgttgaccaagat	*hcdR* gene, primer for qPCR	This study
4hcdRqPCR_R	caaccctgaaccctttgttg	*hcdR* gene, primer for qPCR	This study
4hp1-16qPCR_F	atgccaaggattcaggacag	*hp4* gene, primer for qPCR	This study
4hp1-16qPCR_R	ttgacgtcctcgggataaac	*hp4* gene, primer for qPCR	This study
4hp4-16qPCR_F	actggacgtcgaggaagtgt	*hcdD* gene, primer for qPCR	This study
4hp4-16qPCR_R	gcccctaaccctctcagttc	*hcdD* gene, primer for qPCR	This study
4hp7-16qPCR_F	tctgggtaccgcctacaaag	*hcdE* gene, primer for qPCR	This study
4hp7-16qPCR_R	gccaggtctttcaccttctg	*hcdE* gene, primer for qPCR	This study
Woo1	agagtttgatcmtggctc	16S rRNR gene	[2]
Woo2	gntaccttgttacgactt	16S rRNR gene	[2]

Amplification of genes was conducted using Phusion High-Fidelity PCR Master Mix with HF Buffer, following the user manuals provided by manufacturer of reagents. Amplification conditions:

(a) *xenA38, xenA45* and *xenA205* genes: initial denaturation for 1 min at 98°C, then 30 cycles of denaturation for 10 s at 98°C, annealing for 15 s at 68.5°C, and extension for 60 s at 72°C, final extension for 5 min at 72°C;

(b) 3 kb region of *hcdDEFG* genes: initial denaturation for 1 min at 98°C, then 35 cycles of denaturation for 20 s at 98°C, annealing and extension for 8 min at 72°C, final extension for 7 min at 72°C

(c) *hcdE* gene: initial denaturation for 30 s at 98°C, then 35 cycles of denaturation for 10 s at 98°C, annealing for 10 s at 65°C, and extension for 45 s at 72°C, final extension for 3 min at 72°C

Bacterial strains

Pseudomonas mandelii 7HK4 bacterial strain, capable of using 7-hydroxycoumarin as the sole source of carbon and energy, was selected and identified during previous study [1]. For cloning purposes *E. coli* DH5*α* bacteria (ϕ80 *lac*ZΔM15 Δ(*lacZY-argF*)*U169 deoR rec*A1 *endA1 hsdR*17($r_{K^-}m_{K^+}$) *supE44 thi-1 gyrA96 relA1*) (Thermo Fischer Scientific) were used. *E. coli* BL21 (DE3) bacteria (F^- *ompT gal dcm lon hsdS*$_B$(r_B^- m_B^-) *λ*(DE3) [*lac*I *lac*UV5-T7 gene 1, *ind*1, *sam*7, *nin*5]) (Novagen) were used for gene expression studies.

Bacterial culture media

Mineral medium (pH 7.2): 5 g/L NaCl, 1 g/L $NH_4H_2PO_4$, 1 g/L K_2HPO_4, 0,4 g/L $MgSO_4 \cdot 7H_2O$.

Minimal C-750501 medium (pH 8.0) [3].

LB medium (pH 7.2): 20 g of powder in 1 L of water.

BHI medium (pH 7.4): 37 g of powder in 1 L of water.

For the production of agar plates 15 g of agar powder was added to 1 L of medium.

All media were sterilized for 30 minutes at 121°C, 1 atm.

Preparation of cell-free extracts

Cells were sedimented by centrifugation (3,220 × *g*, 15 min). The biomass was resuspended in 3 ml of 50 mM potassium phosphate buffer (pH 7.2). The cells were disrupted by pulse-mode sonication (3 min duration and 1 s cycles) at 4°C. Cell debris was removed by centrifugation (4°C, 16,100 × *g*, 15 min).

NMR spectra of 3-(2,4-dihydroxyphenyl)propionic acid standard

^{1}H NMR (DMSO-d_6, 400 MHz): δ 11.98 (s, 1H), 9.14 (s, 1H), 8.96 (s, 1H), 6.80 (d, *J* = 8.1 Hz, 1H), 6.26 (d, *J* = 2.4 Hz, 1H), 6.11 (dd, *J* = 8.1, 2.4 Hz, 1H), 2.63 (dd, *J* = 8.5, 6.9 Hz, 2H), 2.40 (dd, *J* = 8.5, 6.9 Hz, 2H);

^{13}C NMR (DMSO-d_6, 100 MHz): δ 174.73, 157.00, 156.23, 130.41, 117.79, 106.33, 102.83, 34.63, 25.36.

Analysis of DNA and protein sequences

DNA and protein sequences were analyzed using VectorNTI Advance 9.0 [4] and MEGA 5.0 [5,6], respectively. The search of homologues was conducted against NCBI database using BLAST [7]. Phylogenetic trees were constructed by MEGA version 5.0 application tool [5,6], using the Neighbor-joining method (N-J) [8] in accordance with the Maximum Composite Likelihood model for nucleotides or Poisson model for amino acids [9].

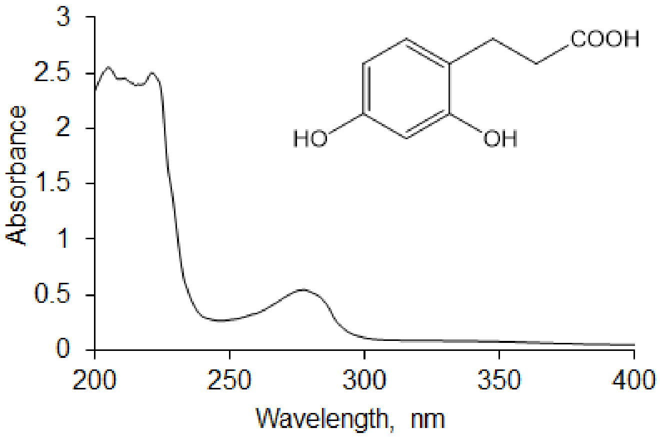

Figure S1. UV-Vis spectrum of 3-(2,4-dihydroxyphenyl)propionic acid.

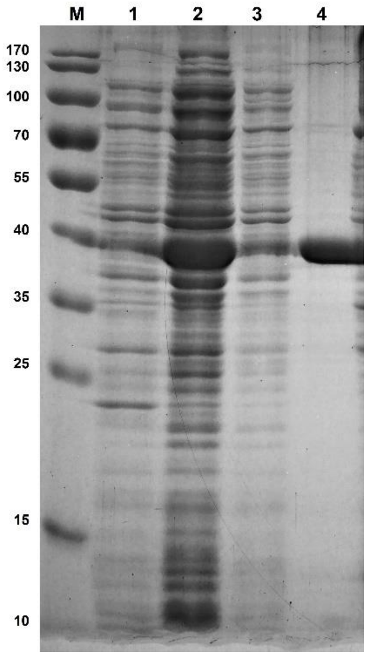

Figure S2. SDS-PAGE of His_6-tagged hcdE protein purified by affinity chromatography. Lane 1 – 3 µl of *E. coli* BL21 cell-free extract without *hcdE* gene, lane 2 – 5 µl of *E. coli* BL21 cell-free extract containing induced recombinant hcdE protein, lane 3 – 5 µl of *E. coli* BL21 cell-free extract containing induced recombinant hcdE protein after purification, lane 4 – 10 µl of eluted His_6-tagged hcdE protein. M – molecular mass ladder (kDa).

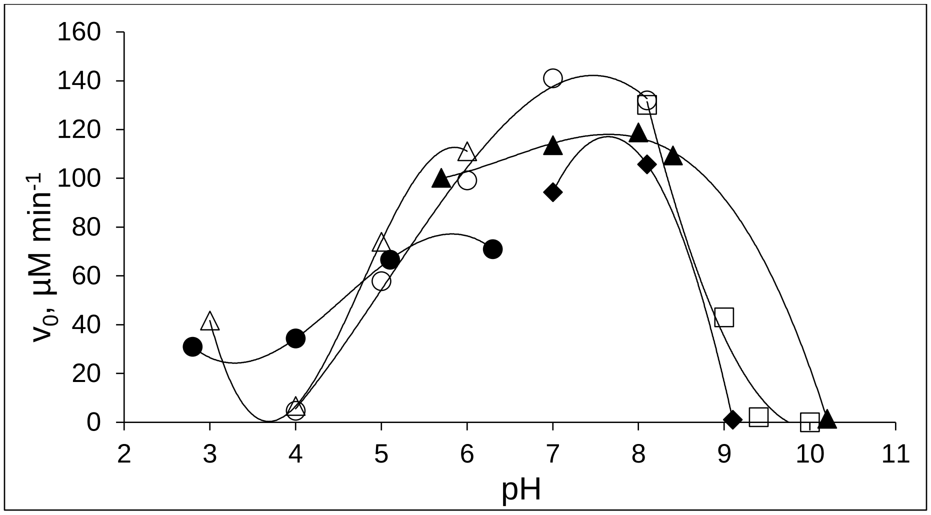

Figure S3. Activity of hcdE protein in different buffer systems and pH. Enzymatic assays were carried out in 50 mM of potassium phosphate (filled triangles), sodium acetate (filled circles), Tris-HCl (filled diamonds), tricine-sodium (empty squares), sodium phosphate and sodium citrate (empty circles) or sodium citrate (empty triangles) buffers with 5 μg of hcdE enzyme and 60 μM of 7-hydroxycoumarin, in presence of 160 μM NADPH at room temperature. Rates of bimolecular reaction were observed at 340 nm wavelength.

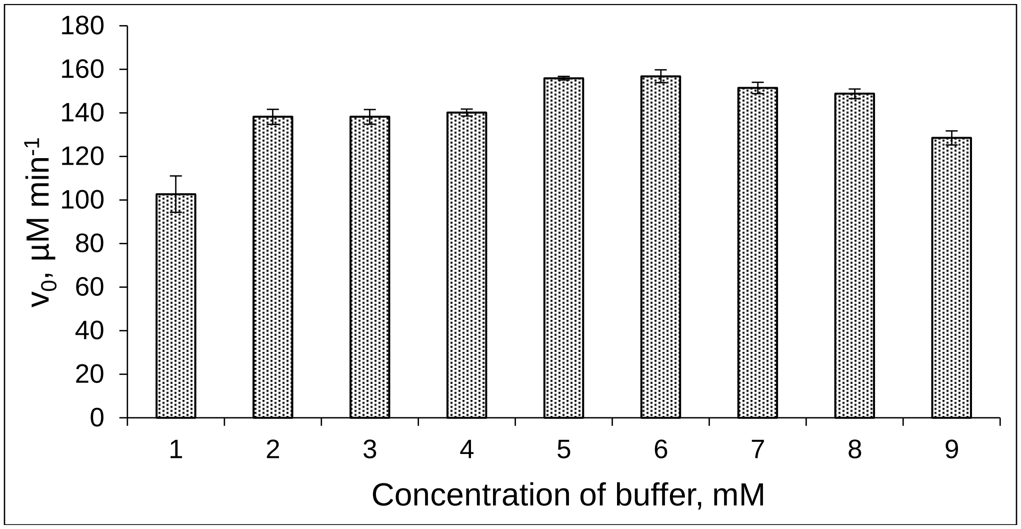

Figure S4. Activity of hcdE protein in buffers of different ionic strength. Enzymatic assays were carried out in 10–150 mM sodium phosphate (Na-P) / sodium citrate (Na-Citr) buffers (pH 7.0) with 5 μg of hcdE enzyme and 60 μM of 7-hydroxycoumarin, in presence of 160 μM NADPH at room temperature. Rates of bimolecular reaction were observed at 340 nm wavelength. Experiment was performed in triplicate and error bars indicate standard error. 1 – 5 mM Na-P and 5 mM Na-Citr; 2 – 10 mM Na-P and 10 mM Na-Citr; 3 – 10 mM Na-P, 10 mM Na-Citr and 10 mM of NaCl; 4 – 25 mM Na-P and 25 mM Na-Citr; 5 – 15 mM Na-P and 35 mM Na-Citr; 6 – 35 mM Na-P and 15 mM Na-Citr; 7 – 25 mM Na-P, 25 mM Na-Citr and 25 mM of NaCl; 8 – 50 mM Na-P and 50 mM Na-Citr; 9 – 25 mM Na-P, 25 mM Na-Citr and 100 mM of NaCl.

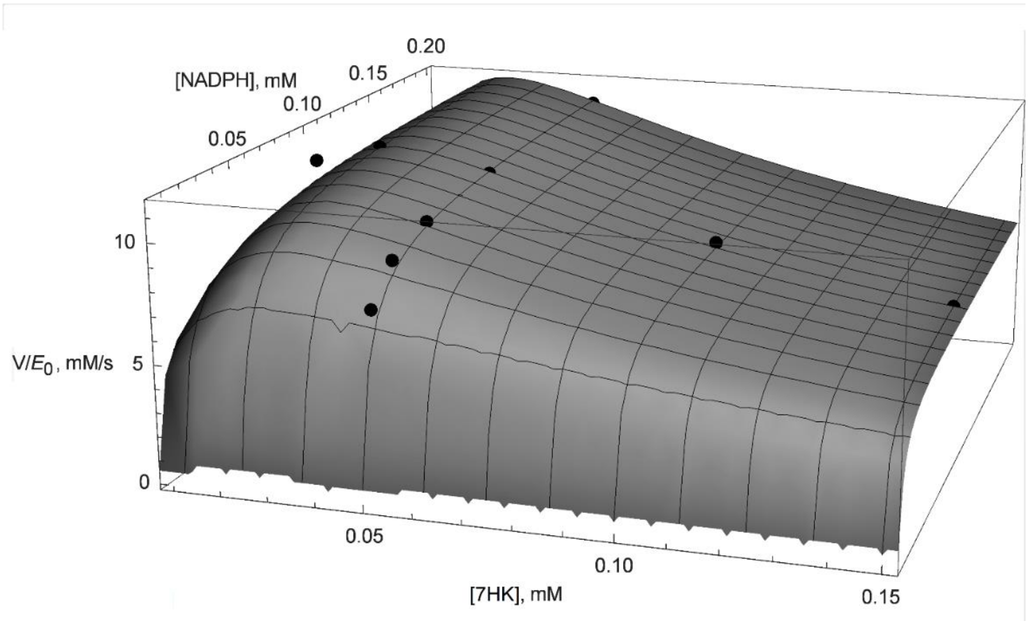

Figure S5. 3D plot of hcdE enzyme kinetic titration data their fit. Enzymatic assays were carried out in 50 mM potassium phosphate (K-P) buffer (pH 7.0) with 5–200 μM NADPH and 5–150 μM 7-hydroxycoumarin at room temperature. HcdE concentration was kept constant at 99 nM. Rates of bimolecular reaction were observed at 365 nm wavelength. Black spheres represent the mean values of three replicates of the initial enzyme rates. The wire frame surface represents the fitted function.

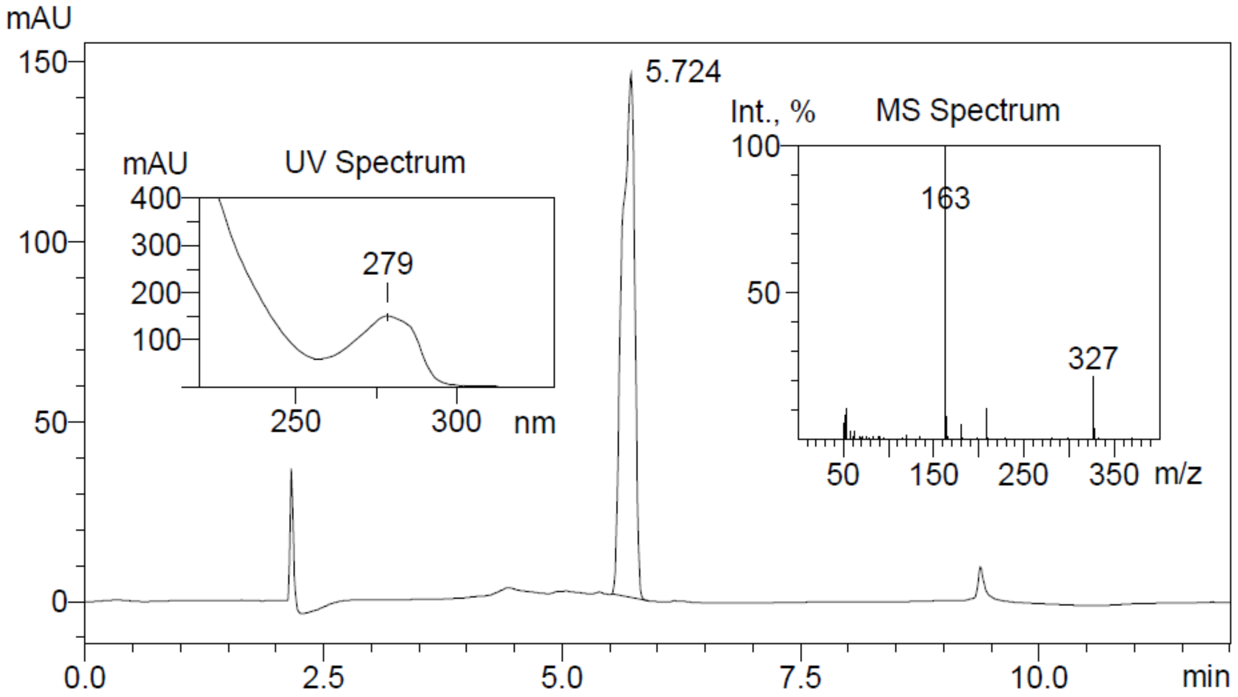

Figure S6. HPLC chromatogram of 7-hydroxy-3,4-dihydrocoumarin. Absorption at 280 nm wavelength was registered. 1 mM of compound was dissolved in acetonitrile. Corresponding UV and MS spectra of the main peak were presented. The negative ions $[M-H]^-$ generated are at m/z 163 (7-hydroxy-3,4-dihydrocoumarin) and 327 (dimer of 7-hydroxy-3,4-dihydrocoumarin).

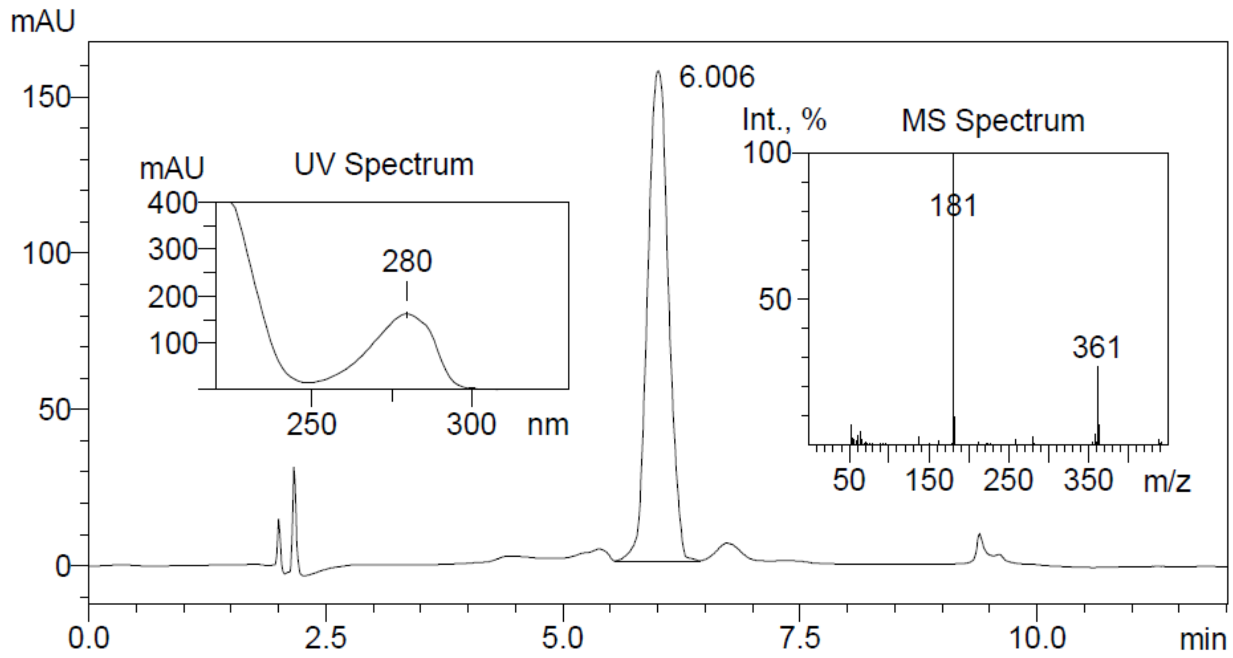

Figure S7. HPLC chromatogram of 3-(2,4-dihydroxyphenyl)propionic acid. Absorption at 280 nm wavelength was registered. 1 mM of compound was dissolved in acetonitrile. Corresponding UV and MS spectra of the main peak were presented. The negative ions $[M\text{-}H]^-$ generated are at m/z 181 (3-(2,4-dihydroxyphenyl)propionic acid) and 361 (dimer of 3-(2,4-dihydroxyphenyl)propionic acid).

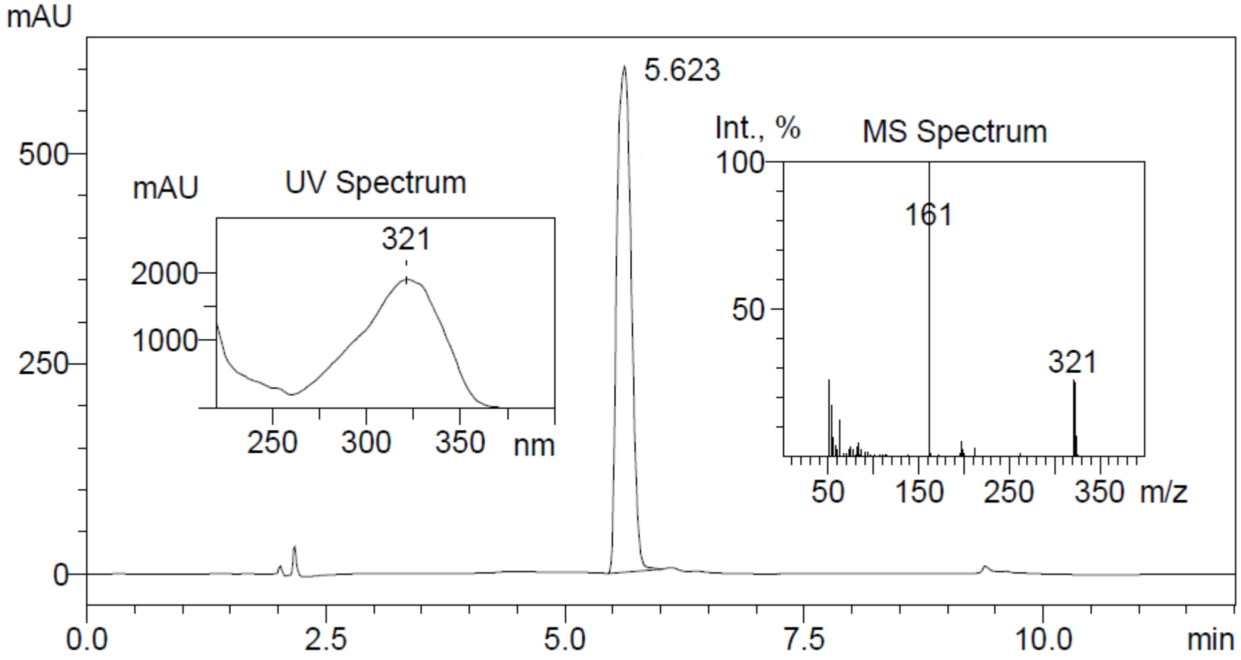

Figure S8. HPLC chromatogram of 7-hydroxycoumarin. Absorption at 280 nm wavelength was registered. 1 mM of compound was dissolved in acetonitrile. Corresponding UV and MS spectra of the main peak were presented. The negative ions $[M\text{-}H]^-$ generated are at m/z 161 (7-hydroxycoumarin) and 321 (dimer of 7-hydroxycoumarin).

References

1. Krikštaponis, A.; Meškys, R. Biodegradation of 7-Hydroxycoumarin in *Pseudomonas mandelii* 7HK4 via ipso-Hydroxylation of 3-(2,4-Dihydroxyphenyl)-propionic Acid. *Molecules* **2018**, 23, doi:10.3390/molecules23102613.
2. Godon, J.J.; Zumstein, E.; Dabert, P.; Habouzit, F.; Moletta, R. Molecular microbial diversity of an anaerobic digestor as determined by small-subunit rDNA sequence analysis. *Appl Environ Microbiol* **1997**, 63, 2802–2813, doi:10.1128/aem.63.7.2802-2813.1997.
3. Sivashanmugam, A.; Murray, V.; Cui, C.; Zhang, Y.; Wang, J.; Li, Q. Practical protocols for production of very high yields of recombinant proteins using *Escherichia coli*. *Protein Sci* **2009**, 18, 936–948, doi:10.1002/pro.102.
4. Gorelenkov, V.; Antipov, A.; Lejnine, S.; Daraselia, N.; Yuryev, A. Set of novel tools for PCR primer design. *BioTechniques* **2001**, 31, 1326–1330, doi:10.2144/01316bc04.
5. Thompson, J.D.; Higgins+, D.G.; Gibson, T.J. CLUSTAL W: improving the sensitivity of progressive multiple sequence alignment through sequence weighting, position-specific gap penalties and weight matrix choice. *Nucleic Acids Research* **1994**, 22, 4673–4680.
6. Tamura, K.; Peterson, D.; Peterson, N.; Stecher, G.; Nei, M.; Kumar, S. MEGA5: Molecular Evolutionary Genetics Analysis Using Maximum Likelihood, Evolutionary Distance, and Maximum Parsimony Methods. *Molecular Biology and Evolution* **2011**, 28(10), 2731–2739, doi:10.1093/molbev/msr121.
7. Altschul, S.F.; Gish, W.; Miller, W.; Myers, E.W.; Lipman, D.J. Basic local alignment search tool. *J Mol Biol* **1990**, 215, 403–410, doi:10.1016/S0022-2836(05)80360-2.
8. Saitou, N.; Nei, M. The neighbor-joining method: a new method for reconstructing phylogenetic trees. *Molecular Biology and Evolution* **1987**, 4, 406–425, doi:10.1093/oxfordjournals.molbev.a040454.
9. Zackerkandl, E.; Pauling, L. Evolutionary Divergence and Convergence in Proteins. *Evolving Genes and Proteins* **1965**, pp. 97–166.
